# EORTC QLQ-C30 general population normative data for Italy by sex, age and health condition: an analysis of 1,036 individuals

**DOI:** 10.1186/s12889-022-13211-y

**Published:** 2022-05-24

**Authors:** Micha J. Pilz, Eva-Maria Gamper, Fabio Efficace, Juan I. Arraras, Sandra Nolte, Gregor Liegl, Matthias Rose, Johannes M. Giesinger, on behalf of the EORTC Quality of Life Group

**Affiliations:** 1 University Hospital of Psychiatry II, Medical University of Innsbruck, Innsbruck, Austria; 2 Innsbruck Institute of Patient-Centered Outcome Research (IIPCOR), Innsbruck, Austria; 3 Health Outcomes Research Unit, Italian Group for Adult Haematologic Diseases (GIMEMA) Data Centre, Rome, Italy; 4 Radiotherapeutic Oncology Department, Complejo Hospitalario de Navarra, Pamplona, Spain; 5 Medical Oncology Department, Complejo Hospitalario de Navarra, Pamplona, Spain; 6 Medical Department, Division of Psychosomatic Medicine, Charité – Universitätsmedizin Berlin, corporate member of Freie Universität Berlin and Humboldt-Universität Zu Berlin, Berlin, Germany

**Keywords:** EORTC QLQ-C30, Italy, Normative values, General population, Health-related quality of life

## Abstract

**Background:**

General population normative values for the widely used health-related quality of life (HRQoL) measure, European Organisation for Research and Treatment of Cancer Quality of Life Questionnaire – Core 30 (EORTC QLQ-C30), are available for a range of countries. These are mostly countries in northern Europe. However, there is still a lack of such normative values for southern Europe. Therefore, this study aims to provide sex-, age- and health condition-specific normative values for the general Italian population for the EORTC QLQ-C30.

**Material and methods:**

This study is based on Italian EORTC QLQ-C30 general population data previously collected in an international EORTC project comprising over 15,000 respondents across 15 countries. Recruitment and assessment were carried out via online panels. Quota sampling was used for sex and age groups (18‍–‍39, 40–49, 50–59, 60–69 and ≥ 70 years), separately for each country.

We applied weights to match the age and sex distribution in our sample with UN statistics for Italy. Along with descriptive statistics, linear regression models were estimated to describe the associations of sex, age and health condition with the EORTC QLQ-C30 scores.

**Results:**

A total of 1,036 respondents from Italy were included in our analyses. The weighted mean age was 49.3 years, and 536 (51.7%) participants were female. Having at least one health condition was reported by 60.7% of the participants. Men reported better scores than women on all EORTC QLQ-C30 scales but diarrhoea. While the impact of age differed across scales, older age was overall associated with better HRQoL as shown by the summary score. For all scales, differences were in favour of participants who did not report any health condition, compared to those who reported at least one.

**Conclusion:**

The Italian normative values for the EORTC QLQ-C30 scales support the interpretation of HRQoL profiles in Italian cancer populations. The strong impact of health conditions on EORTC QLQ-C30 scores highlights the importance of adjusting for the impact of comorbidities in cancer patients when interpreting HRQoL data.

**Supplementary Information:**

The online version contains supplementary material available at 10.1186/s12889-022-13211-y.

## Background

Over recent decades, the importance of health-related quality of life (HRQoL) has steadily increased in oncology research and practice [1]. While there is comprehensive evidence for the validity and reliability of patient-reported outcome (PRO) measures to assess HRQoL, the meaningful and consistent interpretation of such data in clinical trials or in daily clinical practice remains one of the main challenges [2]. Minimal important differences [3, 4], thresholds for clinical importance [5], and normative values [6] are the most important approaches that aid score interpretation. This may be especially true for general population normative values [7], as they can help to identify health issues and support the definition of treatment aims for physicians [8, 9].

Among the standardised PRO measures used to conduct HRQoL assessments, the EORTC QLQ-C30 is the most widely used PRO measure in oncology [10–12]. Acknowledging the variability of normative data that results from cultural and language differences, several sets of country-specific general population normative values of the EORTC QLQ-C30 have been published, mainly investigating the population of central and northern European countries, such as Denmark [8], Germany [13], Norway [14], Slovenia [15], Sweden [16] and The Netherlands [17], leaving most southern European countries, with the exception of Croatia [18], disregarded.

Recently, a large representative online survey was conducted in order to generate general population normative values for 11 European countries, as well as Canada, Russia, Turkey and the US [6]. This study used a uniform sampling and data collection strategy across these countries that provides important advantages for inter-country comparisons. However, although the data provided by this publication supports interpretation of data from multinational projects, the level of detail is not sufficient for informative comparisons of patients against general population data in individual countries.

While sex and age are known to have an impact on HRQoL domains [19], and normative data for these reasons are commonly reported separately for these groups, health conditions frequently found in the general population as well as in cancer populations and cancer survivors have been shown to impact HRQoL to a much larger degree [20–22]. Therefore, a meaningful comparison of specific cancer populations against general population normative data should also account for comorbid health conditions in cancer patients [7].

Given the lack of normative data for the EORTC QLQ-C30 in southern Europe and the need for detailed information on the impact of age, sex and health condition on HRQoL scores, we aimed to provide general population normative values for the EORTC QLQ-C30 for Italy, further stratified by sex, age group, and health condition. This effort supports the meaningful interpretation of PRO scores in clinical research and practice by providing normative data for specific patient groups and, thus, also contributes to setting realistic treatment goals.

## Methods

### The EORTC QLQ-C30 questionnaire

The European Organisation for Research and Treatment of Cancer (EORTC) Quality of Life Questionnaire Core 30 (QLQ-C30) [1] is the most widely used PRO measure in cancer research and practice [10–12]. The EORTC QLQ-C30 consists of 30 items including five functioning scales (physical functioning, social functioning, role functioning, emotional functioning and cognitive functioning), nine symptom scales (fatigue, pain, nausea/vomiting, dyspnoea, sleep disturbances, appetite loss, diarrhoea, constipation and financial difficulties), and a global health status / quality of life (QOL) scale. On the 100-point metric, high scores for functioning scales and the global health status / QOL scale indicate high HRQoL, while high scores on the symptom scales indicate a high symptom burden [1]. Recently, an EORTC QLQ-C30 summary score was developed to complement the individual scale scores of the questionnaires [23, 24]. The Italian version of the EORTC QLQ-C30 has been validated for use in Italian patients [25, 26].

## Data collection

For our analyses, we drew on data collected recently within an EORTC project in 11 European countries, as well as Canada, Russia, Turkey and the US [6]. The panel research company GfK SE was contracted to recruit a representative online sample of 1,000 participants from Italy. Data were collected in March and April 2017. Quota samples were introduced for sex and age groups (18‍‍–‍39, 40–49, 50–59, 60–69 and ≥ 70 years) to obtain at least 100 participants per subgroup. Participants were asked to fill out an online survey containing the EORTC QLQ-C30 and additional information on their sociodemographic characteristics and on current health conditions diagnosed by a medical doctor. GfK SE typically attains response rates between 75 and 90%.

## Statistical analysis

Sample characteristics are given for unweighted data and data weighted to match UN population distribution statistics[27] for the age and sex distribution of the general population in Italy.

General population normative values are given as means and standard deviations (SD) based on the weighted data for groups defined by sex, by age (18–39, 40–49, 50–59, 60–69 and ≥ 70 years), and by health condition (none versus one or more). The percentages of participants obtaining the lowest or highest possible score, i.e. floor and ceiling effects, were calculated for each EORTC QLQ-C30 scale.

In addition, we calculated a multivariable linear regression model to estimate the effects of sex (coding: 0 for female, 1 for male), age (years above 18, linear and quadratic term), and health condition (coding: 0 for none; 1 for one or more health condition(s)); and of the sex-by-age interaction on each EORTC QLQ-C30 scale. This exercise was carried out to allow for a more precise estimation of HRQoL scores than provided in the normative tables. IBM SPSS Version 25 was used for the statistical analysis.

## Results

### Participant characteristics

In the unweighted sample of 1,036 Italian residents, 518 participants (50.0%) were women and the mean age was 52.4 (SD 15.3) years.

Applying weights based on UN statistics [27] increased the proportion of women to 51.7% and decreased the mean age to 49.3 (SD 16.9) years. In the weighted sample, 54.4% of participants had post-compulsory (but below-university level) education, 64.3% were married or in a steady relationship, and 28.4% were working full-time. Having one or more health condition(s) was reported by 60.7% of the participants. The statistical weights applied to the data from individual participants ranged from 0.70 to 2.10 (Table 1).

**Table 1:** Sample characteristics (*N *= 1,036)

		**Unweighted data**	**Weighted data**
Sex N (%)	Male	518 (50.0%)	(48.3%)
	Female	518 (50.0%)	(51.7%)
Age	M (SD)	52.4 (15.3)	49.3 (16.9)
	Median [IQR]	53.5 [25]	50 [29]
Education N (%)	Below compulsory education	0 (0.0%)	(0.0%)
	Compulsory school	17 (1.6%)	(1.5%)
	Some post-compulsory school	122 (11.8%)	(10.9%)
	Post-compulsory below university	565 (54.6%)	(54.4%)
	University degree (Bachelor)	279 (27.0%)	(28.2%)
	Postgraduate Degree	51 (4.9%)	(4.7%)
	Prefer not to answer	2	
Marital status N (%)	Single/not in a steady relationship	214 (20.9%)	(25.5%)
	Married or in a steady relationship	697 (68.1%)	(64.3%)
	Separated/divorced/widowed	113 (11.0%)	(10.3%)
	Prefer not to answer	12	
Employment status N (%)	Full-time employed	299 (28.9%)	(28.4%)
	Part-time employed	76 (7.4%)	(7.5%)
	Homemaker	106 (10.3%)	(9.7%)
	Student	48 (4.6%)	(8.5%)
	Unemployed	94 (9.1%)	(9.7%)
	Retired	272 (26.3%)	(23.2%)
	Self-employed	128 (12.4%)	(12.0%)
	Other	11 (1.1%)	(1.0%)
	Prefer not to answer	2	
Comorbidity N (%)	None	373 (37.7%)	(39.3%)
	One or more	617 (62.3%)	(60.7%)
	Chronic Pain	202 (20.4%)	(19.7%)
	Heart Disease	60 (6.1%)	(5.4%)
	Cancer	19 (1.9%)	(1.7%)
	Depression	98 (9.9%)	(10.2%)
	COPD	23 (2.3%)	(2.0%)
	Arthritis	75 (7.6%)	(7.4%)
	Diabetes	90 (9.1%)	(8.4%)
	Asthma	50 (5.1%)	(5.4%)
	Anxiety disorder	128 (12.9%)	(13.2%)
	Obesity	93 (9.4%)	(9.6%)
	Drug/alcohol disorder	7 (0.7%)	(0.7%)
	Other	165 (16.6%)	(15.5%)
	Prefer not to answer	40	
	Missing	6	

### Normative data for the general Italian population

In Table 2, the general population normative data for the Italian population are presented. The overall mean scores of the functional scales ranged from 73.5 for emotional functioning to 88.1 for social functioning. The highest mean score on the symptom scales was found for fatigue (28.5 points) and the lowest for nausea/vomiting (6.5 points).

**Table 2:** EORTC QLQ-C30 reference values for the general population of Italy

	**All**		**18–39 years**		**40–49 years**		**50–59 years**		**60–69 years**		** ≥ 70 years**	
	*N * = 1,036		*N* = 324		*N* = 192		*N* = 177		*N* = 148		*N * = 195	
	Mean	SD	Mean	SD	Mean	SD	Mean	SD	Mean	SD	Mean	SD
Physical Functioning	85.24	17.02	85.79	18.74	86.50	16.52	86.89	13.91	83.81	15.79	82.69	17.75
Social Functioning	88.05	20.64	87.03	22.71	84.22	23.06	88.51	20.10	90.14	17.50	91.51	16.15
Role Functioning	86.05	22.20	85.63	22.80	85.11	23.56	87.53	20.03	86.11	21.99	86.31	22.01
Emotional Functioning	73.45	22.74	70.23	26.08	68.32	24.32	72.30	19.48	78.67	18.84	80.91	17.65
Cognitive Functioning	86.96	18.63	85.92	21.09	85.11	20.75	87.52	16.90	86.83	17.49	90.09	13.45
Global health status / QOL	64.87	20.33	66.50	20.22	63.11	22.34	62.73	20.06	63.76	20.14	66.67	18.57
Fatigue	28.54	23.86	32.40	25.74	32.04	25.07	26.86	21.35	25.45	21.96	22.58	21.32
Nausea / Vomiting	6.48	15.86	10.14	20.62	9.06	17.23	4.39	11.95	2.58	9.15	2.74	9.49
Pain	20.22	23.93	22.16	24.53	22.73	25.55	18.09	21.94	18.69	23.85	17.62	22.76
Dyspnoea	15.74	23.01	16.56	23.40	18.61	25.38	14.55	20.20	14.61	22.27	13.49	22.74
Insomnia	22.91	27.07	23.42	29.22	28.48	28.48	25.45	26.89	20.76	25.22	15.93	21.50
Appetite loss	8.47	18.96	10.19	22.59	10.84	20.19	7.77	16.66	6.35	15.78	5.54	14.27
Constipation	14.19	23.39	15.15	24.26	17.64	25.86	12.40	22.23	12.46	21.20	12.11	21.64
Diarrhoea	9.29	19.49	12.43	23.71	11.81	20.45	7.61	16.57	6.38	15.52	5.36	14.13
Financial Problems	9.70	21.62	8.27	21.04	12.62	22.63	10.25	22.70	10.47	22.31	8.14	19.81
Summary Score	84.15	14.84	82.47	17.39	81.39	16.18	85.05	12.63	86.02	12.45	87.40	11.19

The mean global health status / QOL score ranged from 62.7 for 50–59-year-old Italians to 66.7 for Italians older than 70 years of age. Furthermore, on the EORTC QLQ-C30 summary score Italians older than 70 years of age reported the highest mean score (87.4 points) across all age groups.

Ceiling and floor effects for the weighted sample are presented in Table 3.

**Table 3:** Floor and ceiling effects in the EORTC QLQ-C30 scales (weighted data)

	Lowest possible score	Highest possible score
	(0 points)	(100 points)
Physical Functioning	0.2%	29.0%
Role Functioning	1.3%	61.7%
Emotional Functioning	0.9%	17.8%
Cognitive Functioning	0.5%	54.6%
Social Functioning	0.6%	67.9%
Global health status / QOL	0.6%	6.2%
Fatigue	21.9%	1.5%
Nausea / Vomiting	79.1%	0.7%
Pain	43.9%	1.0%
Dyspnoea	62.4%	1.7%
Insomnia	50.1%	3.3%
Appetite loss	80.0%	1.3%
Constipation	67.5%	2.3%
Diarrhoea	77.9%	1.2%
Financial Problems	79.4%	2.2%
Summary Score	0.0%	4.2%

### Normative data by sex and age

Table 4 shows general population mean scores for groups defined by sex and age. For male Italians, the lowest (worst) functioning score was found for the age group of 18–39 years on the emotional functioning scale (72.1 points). By contrast, the highest score in the male sample was found for social functioning for those older than 70 years of age (92.8 points). Similarly, Italian women older than 70 years of age displayed the highest score across all age groups on the functioning scales for social functioning (90.6 points). Additionally, emotional functioning showed the poorest functioning scores for Italian women aged between 40 and 49 years (64.1 points).

**Table 4:** EORTC QLQ-C30 reference values for women and men in the general population of Italy

	Men												Women											
	Total		18–39 years		40–49 years		50–59 years		60–69 years		≥ 70 years		Total		18–39 years		40–49 years		50–59 years		60–69 years		≥ 70 years	
	*N* = 500		*N * = 165		*N * = 96		*N* = 87		*N * = 71		*N* = 81		*N* = 536		*N * = 159		*N* = 96		*N* = 90		*N* = 77		*N* = 114	
	Mean	SD	Mean	SD	Mean	SD	Mean	SD	Mean	SD	Mean	SD	Mean	SD	Mean	SD	Mean	SD	Mean	SD	Mean	SD	Mean	SD
Physical Functioning	86.67	17.20	85.47	20.42	88.03	16.77	88.53	13.71	86.14	15.57	86.00	15.25	83.91	16.76	86.13	16.88	84.98	16.22	85.29	14.00	81.67	15.78	80.34	19.04
Social Functioning	88.26	20.49	85.89	23.41	84.63	22.36	91.17	16.67	89.93	18.00	92.83	16.13	87.86	20.80	88.22	21.98	83.82	23.86	85.95	22.72	90.33	17.14	90.57	16.17
Role Functioning	87.14	21.35	84.90	22.35	85.44	23.43	88.67	20.38	88.94	21.43	90.50	16.98	85.04	22.94	86.39	23.30	84.79	23.82	86.44	19.74	83.50	22.32	83.33	24.61
Emotional Functioning	76.15	22.31	72.13	27.09	72.57	22.20	74.58	19.81	81.77	16.90	85.33	13.42	70.94	22.87	68.26	24.91	64.08	25.68	70.10	19.01	75.83	20.15	77.78	19.58
Cognitive Functioning	87.82	18.55	85.35	23.21	87.06	19.24	90.00	14.42	88.45	15.62	90.83	11.71	86.16	18.68	86.51	18.70	83.17	22.09	85.13	18.76	85.33	19.02	89.56	14.59
Global health status / QOL	67.56	19.79	67.94	20.40	66.75	21.29	65.58	18.58	64.85	20.37	72.25	16.86	62.35	20.52	65.00	19.99	59.47	22.88	59.97	21.12	62.75	20.01	62.71	18.79
Fatigue	26.26	23.65	31.93	25.91	29.13	24.81	23.89	20.01	22.44	21.47	17.22	19.19	30.67	23.89	32.88	25.63	34.95	25.12	29.74	22.30	28.22	22.17	26.37	22.01
Nausea / Vomiting	5.73	16.66	10.50	23.29	7.12	16.28	2.67	10.53	2.31	9.16	0.67	4.05	7.18	15.06	9.76	17.50	11.00	18.02	6.05	13.02	2.83	9.21	4.21	11.74
Pain	18.11	22.45	22.56	23.83	22.49	25.33	15.00	20.05	14.19	21.18	10.67	15.82	22.19	25.10	21.75	25.31	22.98	25.90	21.08	23.34	22.83	25.51	22.56	25.55
Dyspnoea	13.98	21.85	16.20	23.98	15.21	22.28	11.33	18.50	14.19	23.31	10.67	18.30	17.37	23.95	16.92	22.86	22.01	27.84	17.65	21.36	15.00	21.42	15.49	25.32
Insomnia	20.18	27.04	22.52	30.40	26.21	29.03	20.33	24.59	16.50	22.96	11.33	20.25	25.46	26.88	24.35	28.03	30.74	27.89	30.39	28.20	24.67	26.68	19.19	21.85
Appetite loss	7.67	18.50	10.96	23.84	8.74	16.81	5.33	14.00	5.28	16.18	4.33	12.24	9.22	19.36	9.40	21.27	12.94	22.99	10.13	18.65	7.33	15.43	6.40	15.55
Constipation	13.14	22.45	14.08	25.30	14.89	22.26	9.67	18.54	11.88	21.95	14.00	20.77	15.16	24.21	16.27	23.16	20.39	28.87	15.03	25.11	13.00	20.63	10.77	22.23
Diarrhoea	9.69	20.24	14.99	26.37	11.65	20.20	5.67	13.46	4.62	13.39	5.33	13.18	8.93	18.77	9.77	20.35	11.97	20.81	9.48	19.00	8.00	17.19	5.39	14.83
Financial Problems	9.03	20.33	8.83	21.74	11.65	21.25	8.33	19.19	9.90	21.93	6.33	15.51	10.33	22.75	7.68	20.33	13.59	24.01	12.09	25.62	11.00	22.78	9.43	22.34
Summary Score	85.48	15.39	82.31	19.41	83.25	15.68	87.62	11.52	87.99	12.26	90.10	8.93	82.90	14.21	82.65	15.06	79.53	16.53	82.57	13.20	84.21	12.43	85.48	12.23

Fatigue and insomnia appeared to be the most prominent symptoms across Italian age and sex groups. Weighted mean scores for fatigue ranged from 17.2 for Italian men older than 70 years of age to 35.0 for Italian women between 40 and 49 years of age. Similarly, mean scores for insomnia ranged from 11.3 for male Italians aged 70 + to 30.7 for female Italians in the 40–49-year-old range.

With very few exceptions, men scored better than women, i.e., higher on the functioning scales and lower on the symptom scales. The same pattern was found for the EORTC QLQ-C30 summary score and the global health status / QOL score. When looking at sex differences within age groups, the highest mean difference was found for pain in those above 70 years of age (10.7 points in men vs 22.6 points in women). The largest sex difference on the functioning scales was found for emotional functioning in the age group of the 40–49-year-olds (72.6 points in men vs 64.1 points in women). For further details please see Table 4.

### Normative data by sex and age, and health condition

Across all sex and age groups, general population normative scores were lower on all functioning scales, the global health status / QOL scale and the summary scores for individuals reporting one or more health conditions. For women, the largest mean differences between participants with and without health conditions were found for global health status / QOL scale (mean difference 21.5 points), pain (mean difference 21.5 points) and fatigue (mean difference 21.1 points) scales. Among men, fatigue (mean difference 15.5 points), global health status / QOL (mean difference 15.4 points) and role functioning (mean difference 15.2 points) showed the highest differences between those with and without health conditions. For further details please see Table 5.

**Table 5:** EORTC QLQ-C30 reference values for general population of Italy by age, sex, and health condition

	Women																							
	18–39 years				40-49 years				50-59 years				60-69 years				70 + years				Total			
	one or more health conditions N = 81		no health condition N = 62		one or more health conditions N = 62		no health condition N = 29		one or more health conditions N = 54		no health condition N = 34		one or more health conditions N = 49		no health condition N = 26		one or more health conditions N = 78		no health condition N = 31		one or more health conditions N = 325		no health condition N = 181	
	Mean	SD	Mean	SD	Mean	SD	Mean	SD	Mean	SD	Mean	SD	Mean	SD	Mean	SD	Mean	SD	Mean	SD	Mean	SD	Mean	SD
Physical Functioning	80.54	19.07	92.04	11.10	80.50	17.66	93.76	7.09	80.55	14.99	92.81	8.56	76.61	15.96	90.51	11.60	76.27	18.78	89.14	16.51	78.92	17.66	91.74	11.36
Social Functioning	82.11	25.05	95.68	13.81	75.87	26.01	98.39	6.61	78.42	25.91	96.93	9.39	84.92	19.61	99.49	2.91	86.76	18.09	98.77	4.44	81.84	23.34	97.41	9.70
Role Functioning	77.88	27.71	95.44	12.10	78.36	26.92	97.31	6.24	79.23	21.68	96.93	9.39	76.19	23.57	95.96	13.26	79.90	25.82	90.12	21.26	78.43	25.45	95.18	13.30
Emotional Functioning	60.14	23.32	80.48	21.87	54.23	24.39	84.14	15.59	62.98	18.44	80.26	14.45	70.50	21.78	84.85	13.48	74.14	20.04	84.88	16.95	64.41	22.86	82.39	17.75
Cognitive Functioning	81.52	20.71	93.22	13.05	78.11	24.32	91.94	13.54	81.15	20.32	90.35	14.85	80.69	21.47	92.42	10.33	88.48	16.08	91.36	10.69	82.36	20.72	92.05	12.68
Global health status / QOL	56.45	21.10	76.57	14.22	51.00	21.51	76.88	16.37	50.55	19.14	74.12	15.50	54.23	18.24	78.28	13.71	57.97	18.22	74.07	15.35	54.45	19.88	75.98	14.86
Fatigue	46.22	22.45	17.86	19.43	42.95	24.78	18.64	18.48	38.43	21.74	16.37	16.52	34.57	22.99	16.50	16.30	30.23	20.37	18.52	24.40	38.69	23.10	17.63	19.15
Nausea / Vomiting	12.65	19.54	5.68	14.45	15.67	20.30	2.69	7.58	9.02	15.41	0.88	5.42	4.23	11.21	0.51	2.91	4.90	12.56	2.47	10.00	9.49	16.94	3.04	10.36
Pain	32.15	27.80	8.38	15.00	31.09	27.22	6.99	12.76	31.69	23.54	5.70	11.17	30.69	26.70	8.59	16.28	26.47	25.93	14.81	23.66	30.28	26.33	8.80	16.19
Dyspnoea	27.34	24.83	6.45	14.57	28.86	29.54	8.60	17.16	25.68	22.30	6.14	13.12	19.05	23.02	6.06	15.56	17.65	24.73	11.11	27.66	23.77	25.40	7.48	17.69
Insomnia	35.10	28.63	10.30	16.65	36.32	27.68	17.20	25.67	34.43	30.44	24.56	24.16	30.69	28.97	15.15	18.89	21.57	22.11	14.81	21.30	31.30	27.82	15.50	21.23
Appetite loss	14.26	26.31	2.72	10.99	19.90	26.00	0.00	0.00	13.66	21.43	5.26	12.34	8.47	15.84	6.06	15.56	8.33	17.62	2.47	8.87	12.95	22.46	3.19	10.88
Constipation	23.52	25.02	8.70	18.17	24.88	32.50	12.90	18.64	16.94	26.99	12.28	22.53	14.81	23.07	10.10	15.63	12.75	23.03	6.17	20.70	18.78	26.55	9.80	19.17
Diarrhoea	12.49	23.23	5.39	16.17	15.42	22.73	6.45	15.93	13.11	22.20	3.51	10.39	11.64	20.05	1.01	5.83	7.84	17.37	0.00	0.00	11.91	21.23	3.67	12.56
Financial Problems	11.72	24.99	2.72	10.99	17.91	27.43	5.38	12.48	18.03	30.81	3.51	10.39	16.40	26.75	1.01	5.83	11.76	23.56	3.70	19.20	14.67	26.45	3.22	12.34
Summary Score	75.27	15.22	91.64	9.34	73.23	16.63	91.70	7.74	76.87	13.21	90.97	7.68	79.60	12.75	92.25	6.85	82.76	12.62	91.07	9.17	77.60	14.58	91.51	8.37

	Men																							
	18–39 years				40–49 years				50–59 years				60–69 years				70 + years				Total			
	one or more health conditions N = 70		no health condition N = 93		one or more health conditions N = 52		no health condition N = 35		one or more health conditions N = 50		no health condition N = 34		one or more health conditions N = 49		no health condition N = 20		one or more health conditions N = 53		no health condition N = 25		one or more health conditions N = 275		no health condition N = 207	
	Mean	SD	Mean	SD	Mean	SD	Mean	SD	Mean	SD	Mean	SD	Mean	SD	Mean	SD	Mean	SD	Mean	SD	Mean	SD	Mean	SD
Physical Functioning	74.30	25.45	94.12	9.22	82.38	18.67	97.19	4.56	83.56	15.33	95.56	6.38	82.10	16.60	95.17	7.38	81.82	16.59	94.19	7.68	80.38	19.60	94.99	7.81
Social Functioning	75.54	27.42	93.77	16.19	76.79	24.15	96.93	8.55	87.93	19.22	95.73	11.31	85.48	20.11	100.00	0.00	90.66	18.55	97.85	8.36	82.76	23.24	95.73	12.70
Role Functioning	72.85	27.45	94.44	10.72	77.08	26.72	99.12	3.78	83.91	24.00	95.30	11.46	84.76	24.44	98.28	5.20	87.63	18.60	95.70	12.19	80.68	25.10	95.91	9.88
Emotional Functioning	61.56	27.96	79.85	23.84	64.14	22.26	85.96	13.17	72.41	20.81	76.92	18.52	79.05	17.36	89.66	11.80	82.83	13.73	89.78	11.97	71.30	22.96	82.58	19.68
Cognitive Functioning	77.24	27.39	91.33	17.60	80.65	21.52	96.93	7.62	87.93	16.18	92.74	11.36	85.95	16.75	94.25	11.24	89.65	12.69	93.55	9.34	83.81	20.64	93.07	13.92
Global health status / QOL	59.81	19.65	74.33	18.92	58.33	21.39	78.51	15.59	58.76	17.99	75.43	15.32	58.57	19.30	80.17	14.96	69.57	15.60	79.57	17.53	61.01	19.26	76.43	17.29
Fatigue	41.99	27.56	24.30	22.20	38.69	25.35	14.62	15.32	29.89	21.17	15.67	15.05	28.57	21.85	7.66	12.37	22.56	20.38	6.45	10.29	32.97	24.67	17.44	19.18
Nausea / Vomiting	16.85	26.97	5.76	19.16	9.82	18.21	1.32	5.99	4.60	13.55	0.00	0.00	2.86	10.64	0.57	3.12	0.51	2.88	1.08	6.01	7.60	18.37	2.98	13.43
Pain	33.51	24.37	14.02	20.01	31.85	26.66	8.33	15.87	20.69	22.61	7.26	12.59	18.81	23.11	3.45	10.41	14.39	17.79	3.23	6.72	24.50	24.25	9.60	16.70
Dyspnoea	28.49	26.41	6.50	16.90	19.64	24.45	7.02	13.79	15.52	20.94	5.13	12.21	19.05	25.82	3.45	10.41	13.13	20.19	6.45	13.44	19.77	24.34	6.06	14.65
Insomnia	32.97	33.58	14.36	25.49	36.31	30.69	13.16	22.67	24.14	24.85	14.53	23.98	19.52	24.48	8.05	17.16	14.14	22.73	5.38	12.51	25.92	29.03	12.47	22.85
Appetite loss	17.93	26.78	5.55	20.06	10.71	16.96	2.63	9.12	8.62	17.19	0.85	5.35	7.14	18.79	1.15	6.24	6.06	14.24	1.08	6.01	10.62	20.22	3.31	14.47
Constipation	23.12	31.68	7.59	16.72	21.43	24.98	5.26	12.33	10.92	19.16	6.84	15.66	16.19	24.64	1.15	6.24	17.68	22.09	5.38	12.51	18.27	25.57	6.17	14.63
Diarrhoea	21.50	31.20	10.43	21.25	14.88	21.96	4.39	11.43	8.62	16.01	1.71	7.46	5.71	15.03	2.30	8.66	7.07	14.95	2.15	8.36	12.26	22.40	6.17	16.17
Financial Problems	18.28	29.14	1.90	9.36	18.45	22.86	0.88	5.41	11.49	22.15	2.56	9.02	13.33	25.07	2.30	8.66	9.09	18.10	1.08	6.01	14.40	24.23	1.77	8.27
Summary Score	72.70	22.49	89.62	12.96	76.75	15.79	93.80	5.14	84.06	13.10	92.63	6.44	84.58	13.05	96.12	4.37	87.46	9.26	95.38	5.61	80.54	16.85	92.16	9.86

	Total																							
	18–39 years				40–49 years				50–59 years				60–69 years				70 + years				Total			
	one or more health conditions N = 151		no health condition N = 155		one or more health conditions N = 115		no health condition N = 64		one or more health conditions N = 104		no health condition N = 67		one or more health conditions N = 98		no health condition N = 46		one or more health conditions N = 132		no health condition N = 56		one or more health conditions N = 600		no health condition N = 389	
	Mean	SD	Mean	SD	Mean	SD	Mean	SD	Mean	SD	Mean	SD	Mean	SD	Mean	SD	Mean	SD	Mean	SD	Mean	SD	Mean	SD
Physical Functioning	77.65	22.39	93.28	10.04	81.35	18.07	95.65	6.03	82.01	15.15	94.19	7.61	79.37	16.43	92.58	10.12	78.52	18.07	91.39	13.45	79.59	18.58	93.48	9.75
Social Functioning	79.07	26.30	94.54	15.26	76.29	25.08	97.58	7.71	83.02	23.30	96.32	10.34	85.20	19.76	99.72	2.17	88.34	18.31	98.36	6.44	82.26	23.28	96.52	11.42
Role Functioning	75.55	27.61	94.85	11.27	77.78	26.72	98.31	5.07	81.49	22.84	96.11	10.44	80.50	24.27	96.99	10.44	83.03	23.40	92.61	17.87	79.46	25.29	95.57	11.59
Emotional Functioning	60.80	25.50	80.10	23.00	58.74	23.86	85.14	14.22	67.54	20.09	78.58	16.58	74.80	20.05	86.98	12.85	77.66	18.21	87.07	15.01	67.57	23.14	82.49	18.78
Cognitive Functioning	79.54	24.05	92.09	15.91	79.27	23.02	94.68	10.89	84.43	18.66	91.55	13.17	83.33	19.33	93.24	10.66	88.95	14.76	92.34	10.08	83.03	20.68	92.60	13.35
Global health status / QOL	58.01	20.44	75.23	17.17	54.33	21.67	77.78	15.84	54.52	18.95	74.78	15.31	56.41	18.81	79.12	14.15	62.67	18.07	76.53	16.44	57.46	19.85	76.22	16.18
Fatigue	44.26	24.95	21.71	21.30	41.01	25.02	16.43	16.80	34.30	21.79	16.02	15.68	31.55	22.51	12.57	15.19	27.12	20.65	13.13	20.19	36.07	23.98	17.53	19.14
Nausea / Vomiting	14.59	23.29	5.73	17.37	13.01	19.51	1.93	6.73	6.88	14.64	0.44	3.82	3.54	10.89	0.54	2.97	3.12	10.07	1.85	8.41	8.62	17.62	3.01	12.08
Pain	32.78	26.19	11.75	18.32	31.44	26.85	7.73	14.46	26.37	23.64	6.49	11.84	24.72	25.54	6.30	14.07	21.57	23.66	9.64	18.94	27.63	25.54	9.22	16.45
Dyspnoea	27.87	25.50	6.48	15.96	24.66	27.61	7.73	15.29	20.77	22.14	5.63	12.58	19.05	24.34	4.90	13.44	15.82	23.02	9.03	22.40	21.93	24.98	6.72	16.13
Insomnia	34.12	30.93	12.73	22.39	36.31	28.96	14.98	23.95	29.45	28.22	19.52	24.42	25.08	27.25	11.99	18.29	18.56	22.58	10.60	18.39	28.83	28.48	13.88	22.13
Appetite loss	15.96	26.50	4.42	17.02	15.72	22.72	1.45	6.85	11.22	19.57	3.05	9.68	7.80	17.31	3.88	12.45	7.41	16.31	1.85	7.69	11.88	21.47	3.25	12.90
Constipation	23.34	28.20	8.04	17.27	23.31	29.25	8.70	15.82	14.03	23.61	9.54	19.43	15.51	23.76	6.12	13.05	14.74	22.70	5.82	17.38	18.55	26.08	7.86	16.98
Diarrhoea	16.66	27.49	8.40	19.47	15.18	22.29	5.31	13.56	10.94	19.49	2.60	9.01	8.66	17.86	1.58	7.17	7.53	16.38	0.96	5.63	12.07	21.76	5.00	14.63
Financial Problems	14.75	27.09	2.23	10.02	18.16	25.35	2.90	9.47	14.87	27.04	3.03	9.66	14.86	25.83	1.58	7.17	10.68	21.48	2.53	14.78	14.55	25.43	2.45	10.38
Summary Score	74.08	18.91	90.43	11.65	74.83	16.28	92.86	6.47	80.35	13.58	91.81	7.08	82.10	13.08	93.97	6.13	84.66	11.58	92.99	8.02	78.95	15.72	91.86	9.19

### Regression models for prediction of normative scores

To allow for the calculation of age-, sex- and health condition-specific normative data, we provide a supplementary table with regression coefficients for each of these characteristics for the individual EORTC QLQ-C30 scales (variable coding is given above).

For illustration, please find below the calculation of a normative social functioning score for a 45-year-old Italian woman with a health condition based on the regression model:

Social Functioning (predicted) = 93.54 + sex * 5.29 + (age-18) * -0.13 + (age-18)^2^ * 0.006 + (age—18) * sex * -0.17—health condition * 15.36.

Social Functioning (predicted) = 93.54 + 0 * 5.29 + (45–18) * -0.13 + (45–18)^2^ * 0.006 + (45—18) * 0 *—0.17—1 * 15.36 = 79.04.

## Discussion

As part of this study, we established normative data for the EORTC QLQ-C30 for the general Italian population, separately for groups defined by sex, age and health condition, to facilitate interpretation of EORTC QLQ-C30 data in clinical research and practice. A detailed depiction of various general population subgroups was provided, thus allowing healthcare professionals and researchers to utilise the most accurate approximation when interpreting HRQoL results of Italian cancer patients. Additionally, we provided regression equations, facilitating the calculation of normative values for specific subgroups.

When scrutinising these normative values, three main findings were observed. First, the elderly Italian population tended to experience higher HRQoL, shown for example by the summary score, compared to the younger age groups. This is in line with the results of a previous study completed in Australia [28] but in contrast to other European normative data [13, 16, 18]. Second, men reported higher levels of functioning and lower symptom burden than women, for all scales but one. Such sex differences have been reported repeatedly in studies collecting general population normative data [29] and in the literature concerning cancer patients [19, 30]. While in our data sex differences favouring men were observed for nearly all scales, there is substantial variation across countries, with, for example, a Danish study observing such differences only for one-third of the EORTC QLQ-C30 scales [8] and a recent German study reporting such for about two-thirds of the scales [31].

However, age and sex differences were rather small compared to those between participants with and without health conditions. The large impact of health conditions on EORTC QLQ-C30 scores is in line with previous literature [8, 29] and highlights the importance of adjusting normative scores for cancer populations for the presence of other health conditions (comorbidities) when interpreting scores. In our analysis, we covered a range of common health conditions likely to have an impact on EORTC QLQ-C30 scores with the additional possibility for patients to report any other condition that was diagnosed by a doctor. Unlike other studies [32–34], we did not rely on the Charlson Comorbidity Index [35], as its selection of included conditions was made to predict survival, and as a result it covers very severe health conditions, with mostly low prevalence rates. In contrast, our assessment of health conditions covered less life-threatening diseases, with higher prevalence but a presumably strong impact on HRQoL, including chronic pain, depression, anxiety disorders and obesity, among others. Given the large impact on HRQoL observed in our study, we encourage future assessments of health conditions to take a wider perspective than the set of conditions included in the Charlson Comorbidity Index, if the interest is in patients’ HRQoL rather than survival.

In clinical practice, this general population normative data may provide clinicians with realistic treatment goals in cancer patients with good prognosis undergoing curative treatment, and in patients during cancer rehabilitation. In cancer survivors it may allow the identification of HRQoL domains that continue to be impaired after successful treatment. The choice of the most appropriate comparator group for an individual patient or patient group is crucial for meaningful interpretation of scores. For example, thyroid cancer patients experience compromised HRQoL prior to [36], during [37] and after treatment [38]. After treatment completion normative data from the general population may be the most appropriate comparator, as it can be expected that a large proportion of patients return to pre-disease HRQoL levels. However, during treatment, reference values from patients with the same disease and treatment, or thresholds for clinical importance [5], may be more relevant for score interpretation.

Furthermore, pre-treatment data, i.e. data collected between diagnosis and start of treatment, is frequently missing, and even if collected will not reflect pre-disease levels since the distress of the diagnosis itself and early disease symptoms possibly preceding diagnosis will lower HRQoL. We argue that general population data may be considered to reflect pre-disease levels and may serve as a kind of baseline for interpreting trajectories of disease and treatment burden.

Strengths of this study include the detailed comparisons between population subgroups and an analytical procedure that is in accordance with previous studies [6, 39]. One of the limitations of this study is the online data collection from the general Italian population. This may lead to a selection bias, as people who are computer illiterate or do not have access to the internet are a priori excluded from this study. This effect may be especially relevant for the elderly and/or financially disadvantaged population. Additionally, we were not able to provide further analyses concerning elderly people, as ≥ 70 years was the highest age group recorded. For the Italian population, with an average life expectancy of 83.4 years – amongst the highest in the world [40] – a more differentiated perspective concerning this group is desirable in future studies. Lastly, the binary coding of existing health conditions might be a limitation of this study. While we simplified the coding and therefore enhanced the applicability of the normative scores in clinical practice and research, information on the increasing negative impact of accumulating health conditions is lost. This issue should be addressed in future research.

## Conclusion

In conclusion, our data will facilitate the interpretation of the EORTC QLQ-C30 in Italian cancer patients at both the individual patient and the group level. It may also lead to more valid conclusions when comparing Italian cancer patients against patients from other countries. Given the major impact of health conditions on HRQoL, comorbidities should be considered when evaluating EORTC QLQ-C30 scores from cancer patients.

## Supplementary Information

**Additional file 1: Supplementary Table S1:** Regression models for the EORTC QLQ-C30 values in the General Population of Italy.

## Data Availability

The data that support the findings of this study are available from the EORTC but restrictions apply to the availability of these data, which were used under license for the current study, and so are not publicly available. Data are however available from the authors upon reasonable request and with permission of Sandra Nolte.

## Abbreviations

HRQoL: Health-related quality of life
PROs: Patient-reported outcomes
QOL: Quality of life
EORTC: European Organisation for Research and Treatment of Cancer
QLQ-C30: Quality of Life Questionnaire Core 30
SD: Standard Deviation
