# Supplementary material for: EORTC QLQ-C30 general population normative data for Italy by sex, age and health condition: an analysis of 1,036 individuals
Source: BMC Public Health. 2022 May 24;22:1040. doi: 10.1186/s12889-022-13211-y (PMC9128281; doi:10.1186/s12889-022-13211-y)
Supplement: Supplementary file 1 — Additional file 1: Supplementary Table S1: Regression models for the EORTC QLQ-C30 values in the General Population of Italy. [file 12889_2022_13211_MOESM1_ESM.docx]

Supplementary Table S1: Regression models for the EORTC QLQ-C30 values in the General Population of Italy

|  | Intercept | Sex^1^ |  | Age^2^ |  | Age* squared^3^ |  | Age*-by-sex^4^ |  | Health condition^5^ |  | Adjusted R Square |
| --- | --- | --- | --- | --- | --- | --- | --- | --- | --- | --- | --- | --- |
|  |  | Coeff. | p-value | Coeff. | p-value | Coeff. | p-value | Coeff. | p-value | Coeff. | p-value |  |
| Physical Functioning | 89.28 | 3.60 | 0.089 | 0.35 | 0.005 | -0.004 | 0.021 | -0.18 | 0.002 | -13.96 | <0.001 | 0.17 |
| Role Functioning | 90.17 | 6.50 | 0.022 | 0.23 | 0.158 | 0.003 | 0.877 | -0.26 | 0.001 | -16.80 | <0.001 | 0.13 |
| Emotional Functioning | 83.15 | -0.91 | 0.746 | -0.43 | 0.009 | 0.013 | <0.001 | -0.12 | 0.128 | -16.28 | <0.001 | 0.17 |
| Cognitive Functioning | 91.63 | 2.82 | 0.248 | -0.16 | 0.263 | 0.006 | 0.010 | -0.14 | 0.047 | -10.29 | <0.001 | 0.08 |
| Social Functioning | 93.54 | 5.29 | 0.043 | -0.13 | 0.409 | 0.006 | 0.010 | -0.17 | 0.019 | -15.36 | <0.001 | 0.14 |
| Global health status / QOL | 79.35 | 1.92 | 0.438 | -0.37 | 0.010 | 0.009 | <0.001 | -0.19 | 0.005 | -18.91 | <0.001 | 0.22 |
| Fatigue | 25.31 | -2.88 | 0.324 | -0.21 | 0.217 | -0.004 | 0.184 | 0.22 | 0.007 | 20.39 | <0.001 | 0.20 |
| Nausea / Vomiting | 8.62 | -1.87 | 0.367 | -0.15 | 0.214 | -0.002 | 0.291 | 0.11 | 0.062 | 6.94 | <0.001 | 0.08 |
| Pain | 14.18 | -8.46 | 0.005 | -0.02 | 0.902 | -0.005 | 0.044 | 0.38 | <0.001 | 19.57 | <0.001 | 0.17 |
| Dyspnoea | 6.68 | 0.17 | 0.954 | 0.15 | 0.384 | -0.005 | 0.049 | 0.10 | 0.249 | 15.78 | <0.001 | 0.12 |
| Insomnia | 11.46 | -1.83 | 0.600 | 0.53 | 0.010 | -0.014 | <0.001 | 0.21 | 0.033 | 15.88 | <0.001 | 0.11 |
| Appetite loss | 6.52 | -2.93 | 0.242 | -0.03 | 0.830 | -0.003 | 0.202 | 0.14 | 0.041 | 9.49 | <0.001 | 0.07 |
| Constipation | 7.30 | 3.94 | 0.203 | 0.15 | 0.412 | -0.004 | 0.127 | -0.06 | 0.471 | 11.28 | <0.001 | 0.06 |
| Diarrhoea | 10.69 | -7.97 | <0.001 | 0.00 | 0.989 | -0.005 | 0.035 | 0.22 | 0.002 | 8.49 | <0.001 | 0.07 |
| Financial Problems | 0.95 | -3.56 | 0.207 | 0.29 | 0.076 | -0.007 | 0.010 | 0.14 | 0.082 | 12.37 | <0.001 | 0.08 |
| Summary Score | 89.00 | 3.01 | 0.092 | -0.04 | 0.690 | 0.005 | 0.004 | -0.17 | 0.001 | -13.89 | <0.001 | 0.23 |
| ^1^ sex (coding: 0 for female; 1 for male)  ^2^ age (years above 18)  ^3^ age (years above 18) quadratic term  ^4^ sex-by-age interaction  ^5^ health conditions (coding: 0 for none; 1 for one or more health condition(s)) | | | | | | | | | | | |  |
